# A survey of organizational structure and operational practices of elite youth football academies and national federations from around the world: A performance and medical perspective

**DOI:** 10.3389/fspor.2022.1031721

**Published:** 2022-11-23

**Authors:** Warren Gregson, Christopher Carling, Antonio Gualtieri, James O'Brien, Patrick Reilly, Francisco Tavares, Daniele Bonanno, Emmanuel Lopez, Joao Marques, Lorenzo Lolli, Valter Di Salvo

**Affiliations:** ^1^Aspire Academy, Football Performance & Science Department, Doha, Qatar; ^2^Football Exchange, Research Institute of Sport Sciences, Liverpool John Moores University, Liverpool, United Kingdom; ^3^French Football Federation Research Centre, French Football Federation, Clairefontaine National Football Centre, Clairefontaine-en-Yvelines, France; ^4^Laboratory Sport, Expertise and Performance (EA 7370), French Institute of Sport (INSEP), Paris, France; ^5^Sport Science and R&D Department, Juventus FC, Turin, Italy; ^6^School of Health and Sports Science, University of Suffolk, Ipswich, United Kingdom; ^7^Red Bull Athlete Performance Center, Salzburg, Austria; ^8^Premier League, London, United Kingdom; ^9^Medical and Performance Department, Sporting Clube de Portugal, Lisbon, Portugal; ^10^Faculty of Medicine, Rehabilitation and Functional Performance Program, University of São Paulo, São Paulo, Brazil; ^11^Aspetar, Rehabilitation Department, Qatar Orthopedic and Sports Medicine Hospital, Doha, Qatar; ^12^Department of Movement, Human and Health Sciences, University of Rome “Foro Italico”, Rome, Italy

**Keywords:** player development, strategy, process management, knowledge management, decision-making, research, innovation

## Abstract

**Aim:**

Medical and performance units are integral components of player development programmes in elite football academies. Nevertheless, the nature of the operational processes implemented by practitioners within clubs and national federations remains unexplored. The aim of the present study, therefore, was to survey elite youth professional football academies from around the world regarding the operational processes adopted by their medical and performance units.

**Methods:**

Of the 50 organizations invited, 10 national federations and 25 clubs took part in the survey resulting in a response rate of 70% (95% confidence interval, 56%−81%). The respondents represented three groups: senior club and academy management, performance, and medical staff.

**Results:**

The majority (60%−90%) of clubs and national federations reported strategic alignment between senior and academy medical and performance units as well as between academy medical and performance units. Survey responses indicated substantial heterogeneity in the composition and number of medical and performance professionals employed in academies. The majority of respondents agreed their medical and performance departments were effective in utilizing staff knowledge and external sources of knowledge to inform their practice (56%−80%). Performance staff (40%−50%) and physiotherapists (30%−32%) were deemed most influential in injury prevention programmes. During the return-to-play process, the influence of specific practitioners in the medical and performance units was dependent upon the phase of return-to-play. Shared decision-making was common practice amongst performance and medical staff in injury prevention and return-to-play processes. Medical and performance data were generally centralized across the first team and academy in majority (50%−72%) of clubs and national federations. Data were integrated within the same data management system to a higher degree in clubs (68%) vs. national federations (40%). Research and development activity were reported for most academies (50%−72%), and generally led by the head of performance (37%) or team doctor (21%). Research activities were largely undertaken *via* internal staff (~100%), academic collaborations (50%−88%) and/or external consultants and industry partnerships (77%−83%) in the national federation and clubs.

**Conclusion:**

Collectively, these findings provide a detailed overview regarding key operational processes delivered by medical and performance practitioners working in elite football academies.

## Introduction

Professional football clubs and national football federations must remain competitive on both a sport and financial level in order to be effective and successful within the growing sports industry (1, 2). The combined efforts of all relevant stakeholders will impact the ability of clubs and national federations to achieve their strategic objectives (1, 3). In this context, the organization may benefit from enhanced performance of their stakeholders through more effective and efficient management practices, with a particular reference to organizational structure and operational processes (2–6). Given the significant cost of recruiting elite footballers in the transfer market, investment in youth academies, talent identification and development is increasing in strategic importance for many football clubs around the world (7). As key stakeholders in the long-term development of players, the effectiveness of academy and national federation medical and performance departments is, therefore, likely to gain in importance for maximizing the player development (7, 8).

Frameworks for athlete development typically attempt to combine both best practice and experience underpinned by high-quality up-to-date research (9, 10). Research in football generally seeks to provide evidence regarding the value and application of business solutions addressing technical, tactical, physical, and psychological components of football performance, thereby supporting practitioners to make evidence-based decisions on the practices they may employ within an applied setting (8). Conversely, with concrete characterizations provided by research from other sports in mind (6, 11), there is limited information regarding the organizational structures and operational practices adopted by medical and performance departments as they strive to maximize their service provision support to players (2, 8). It seems logical to assume diversity in the strategies applied in practice within modern football organizations across the world, and probably due in part to differences in financial resources as well as cultural influences across different countries (2). However, an understanding of common professional practices is of interest to the clubs, federations, individual practitioners, and researchers since it can support identifying specific areas that may warrant further scrutiny as part of strategies adopted by football organizations to drive competitive advantage.

We therefore aimed to gather information regarding the organizational structure and operational practices of medical and performance departments in elite youth academies and national football federations from around the world. Specifically, we intended to provide a detailed overview in relation to the following areas of elite practice: strategy and structure, knowledge management processes, injury prevention and return-to-play processes, data management processes and research and development activities.

## Materials and methods

### Survey design and distribution

We conducted a cross-sectional survey to gather information on perceptions of staff members regarding organizational structure and operational practices at their club academies. The survey was developed by a panel of 10 experts with five or more years of experience working in professional football at European and Middle-Asian youth academies. Published work in this (2, 12–14) and other research fields (4) informed the survey design (Table 1) consisting of questions covering specific areas: (1) background information (six items), (2) academy strategy (five items), (3) academy structure (nine items) [5], (4) knowledge management (eight items) [5], return to play (seven items), injury prevention (nine items), data management (six items), and research and development (five items), respectively. The underlying structure of club and federation academies was examined according to the organizational structure frameworks described by Steiger et al. (4) and Mintzberg (15). The return-to-play process was broken down into specific sub-phases (16). The return- to-training sub-phase refers to the gradual re-introduction of the injured player from non-contact to resuming full team training. The return-to-competition sub-phase involves the player's progression in terms of competitive match minutes, whereas in return-to-performance sub-phase the player is deemed to meet the required competition demands (16).

**Table 1:** The types of questions used in the survey.

**Question type**	**Definition**
Multiple choice	Choose one answer from list of answers
Simple multiple choice	Choose one answer from list of two: yes or no
Checkboxes	Select multiple answers from list of answers
Single textbox	Write numerical answer to question
Ranking	Rank a list of options in order of preference using a numeric dropdown list
Matrix/rating scale	Evaluate one or more items using a Likert rating scale (1–7) to assign weights to each answer

The final survey version (Supplementary File 1) was reviewed for content validity by a group involving two practitioners having previously worked in two English Premier League clubs and one academic with expertise in this area of research. Questions involved multiple choice, simple multiple choice (yes/no), checkbox, numerical, or ranking formats (Table 1). The survey was created using the online software SurveyMonkey^®^ (Momentive Inc., USA), and disseminated to organization representatives *via* an email containing instructions on survey purpose, followed by instructions for survey completion. Importantly, respondents were advised to have relevant information available prior to taking part in the survey. In this context, fifty members of a community of practice led by the lead institution (Aspire Academy) were contacted to take part in the present study (7). Of these, 35 practitioners from individual clubs (*n* = 25) and national federations (*n* = 10) agreed to participate in the survey. The 25 clubs were from Europe (*n* = 19; Austria, Belgium, England, France, Italy, Netherlands, Portugal, Russia, Spain, Switzerland, Ukraine), North America (*n* = 2; United States of America), South America (*n* = 3; Argentina, Brazil, Chile), and Africa (*n* = 1; Tunisia). The 10 national federations were from Europe (*n* = 3; England, France, Italy), North America (*n* = 2; Honduras, Mexico), South America (*n* = 2; Argentina, Chile), Africa (*n* = 1; South Africa), and Asia (*n* = 2; Qatar, South Korea). The survey opened on 16/11/2020 and closed on 24/02/2021. This study was approved by the Aspire Zone Foundation Institutional Review Board, Doha, State of Qatar (protocol number: E202007005).

### Statistical analysis

Results were presented as descriptive statistics (17) by organization type. Frequency analysis was conducted for participant characteristics, multiple choice, checkboxes, ranking, Likert-type, and rating scale questions, with the results presented as percentage of respondents and frequency count. The response rate was determined as the number of clubs and national federations respondents who answered by the total number of organizations we invited to take part in the survey, with the Agresti-Coull method (18) used to describe the uncertainty in this estimate expressed as an approximate 95% confidence interval (95% CI). Qualitative terms were also assigned to determine the magnitude of the observed frequencies as follows: All = 100% of respondents; Most = ≥75%; Majority = 55%−75%; Approximately half = ~50%; Approximately a third = ~30%; Minority = <30% (7). Responses involving a numerical answer in single questions (i.e., count data) were presented as median plus interquartile range (IQR) or minimum and maximum values. All statistical analyses were performed using R (version 3.6.3, R Foundation for Statistical Computing).

## Results

### Respondents

Of the 50 organizations invited, 10 national federations and 25 clubs agreed to take part in the survey. The response rate was 70% (95%CI, 56%−81%). Among the 25 club respondents, three were technical directors, three academy directors, three director/head of medical and performance, 11 director/head of performance, one medical doctor, three strength and conditioning coaches and one psychologist. Club respondents had worked at their current clubs for 5 years (IQR, 2–9 years), with 10 years (IQR, 6–18 years) of experience in professional football. Of these, 20% had a PhD degree, 40% a Master degree, and 28% a Bachelor degree.

Amongst the 10 national federation respondents, two were heads of performance, three team coordinators, one medical doctor, one sport scientist, two strength and conditioning coaches, and 1 head of talent identification. Half of the respondents had a PhD degree, 13 years (IQR, 6–22 years) of experience in professional football, and 3.5 years (IQR, 1–5 years) spent at their respective organization.

### Strategy

**Figure 1:**
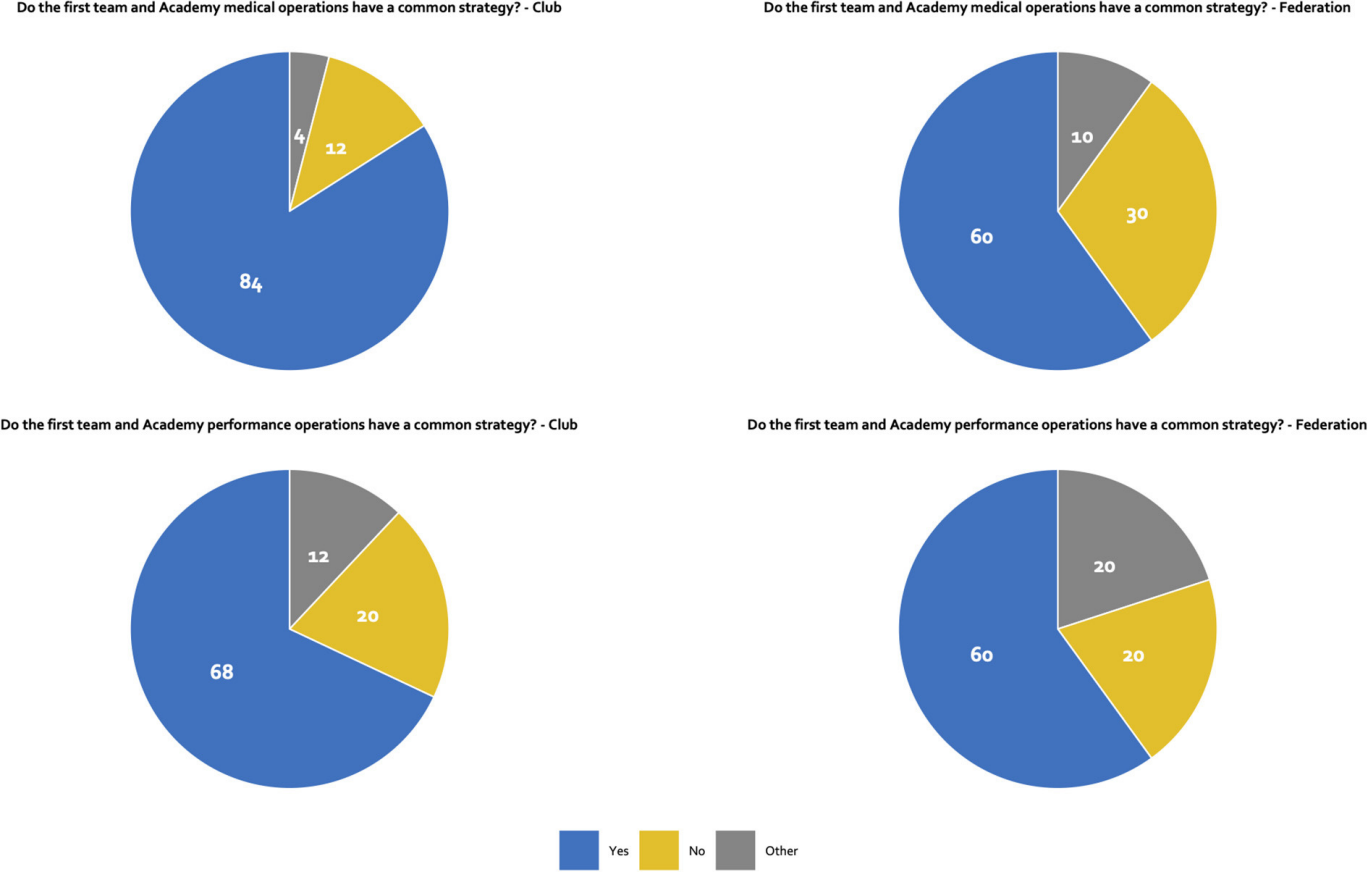
Strategy.

The majority of the club respondents stated that the first team and academy medical (84%) and performance (68%) operations are strategically aligned, with medical staff overseeing academy medical operations reporting to those overseeing the first team (Figure 1). Almost all clubs stated that academy medical and performance strategies are aligned (~90%), with only a third of the clubs' medical and performance operations led by the same individual (~40%).

The majority (60%) of national federations respondents indicated alignment between the first team and academy medical and performance operations (Figure 1). Likewise, staff members overseeing the medical strategy report to first team staff in most of the national federations (~60%). Almost all respondents indicated an alignment between medical and performance strategies (~90%), with the same individual leading both areas in ~50% of the federations surveyed.

### Structure

**Figure 2:**
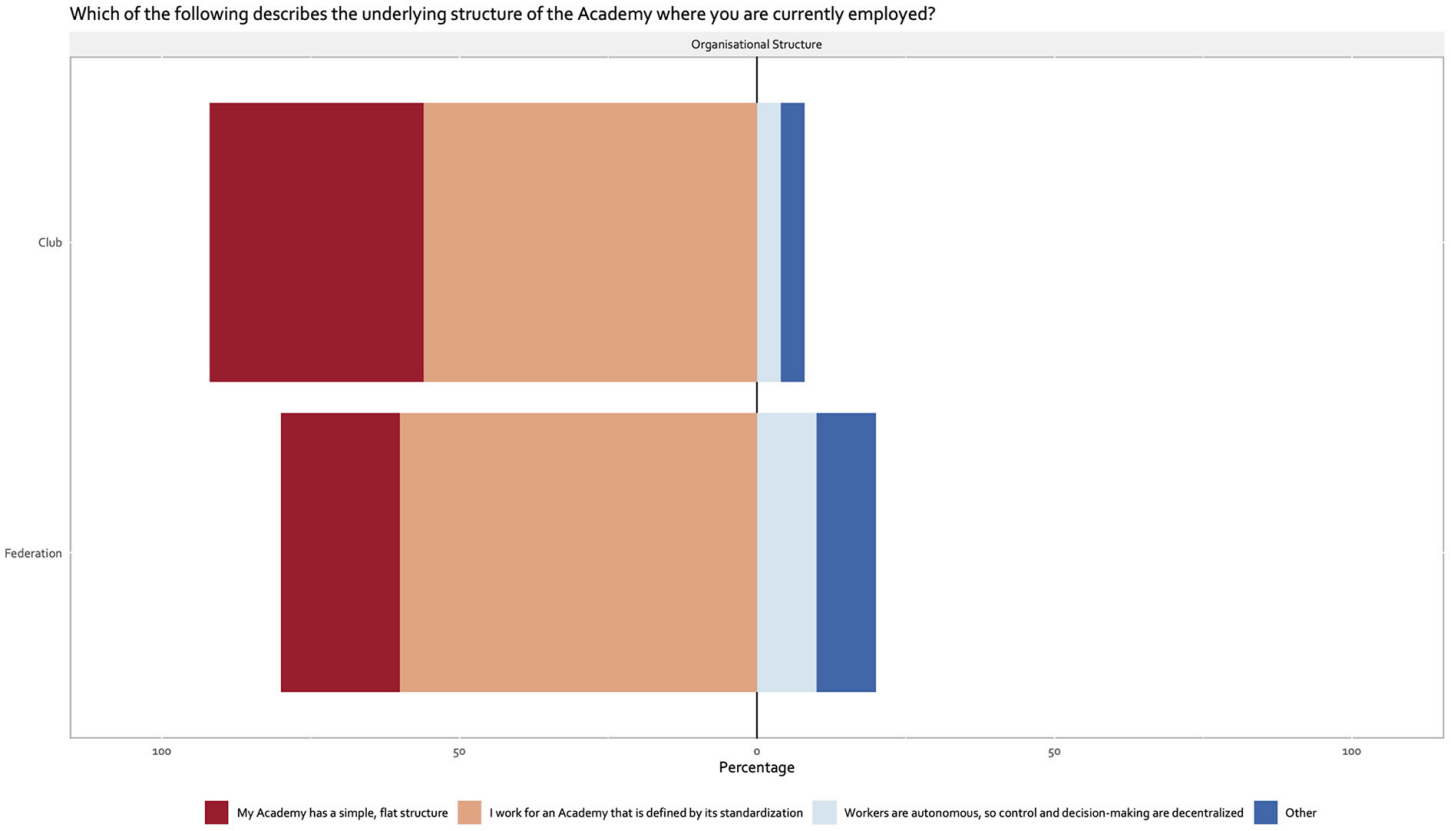
Organizational structure.

The majority (~60%) of club and national federation respondents agreed with the statement their academies' organization “is defined by its standardization. Work is very formalized, there are many routines and procedures, decision-making is centralized, and tasks are grouped by professional departments. Our roles are clearly defined. We have a formal planning process with budgets and audits, (internal or external) and procedures are regularly appraised for efficiency” (Figure 2). In clubs, there were a median of 12 (IQR, 6–16) medical and 9 (IQR, 6–15) performance staff members. In the case of national federations, the median numbers were 10 (IQR, 6–15) and 6 (IQR, 3–24) respectively. The number of medical and performance staff members by employment type and player age category (team) distribution are summarized in Supplementary Tables 1, 2. Organizational structures were in place for 5 years (IQR, 2–8 years) and 8 years (IQR, 5–12) and reviewed on yearly basis in the majority of clubs and national federations, respectively.

### Knowledge management

**Figure 3:**
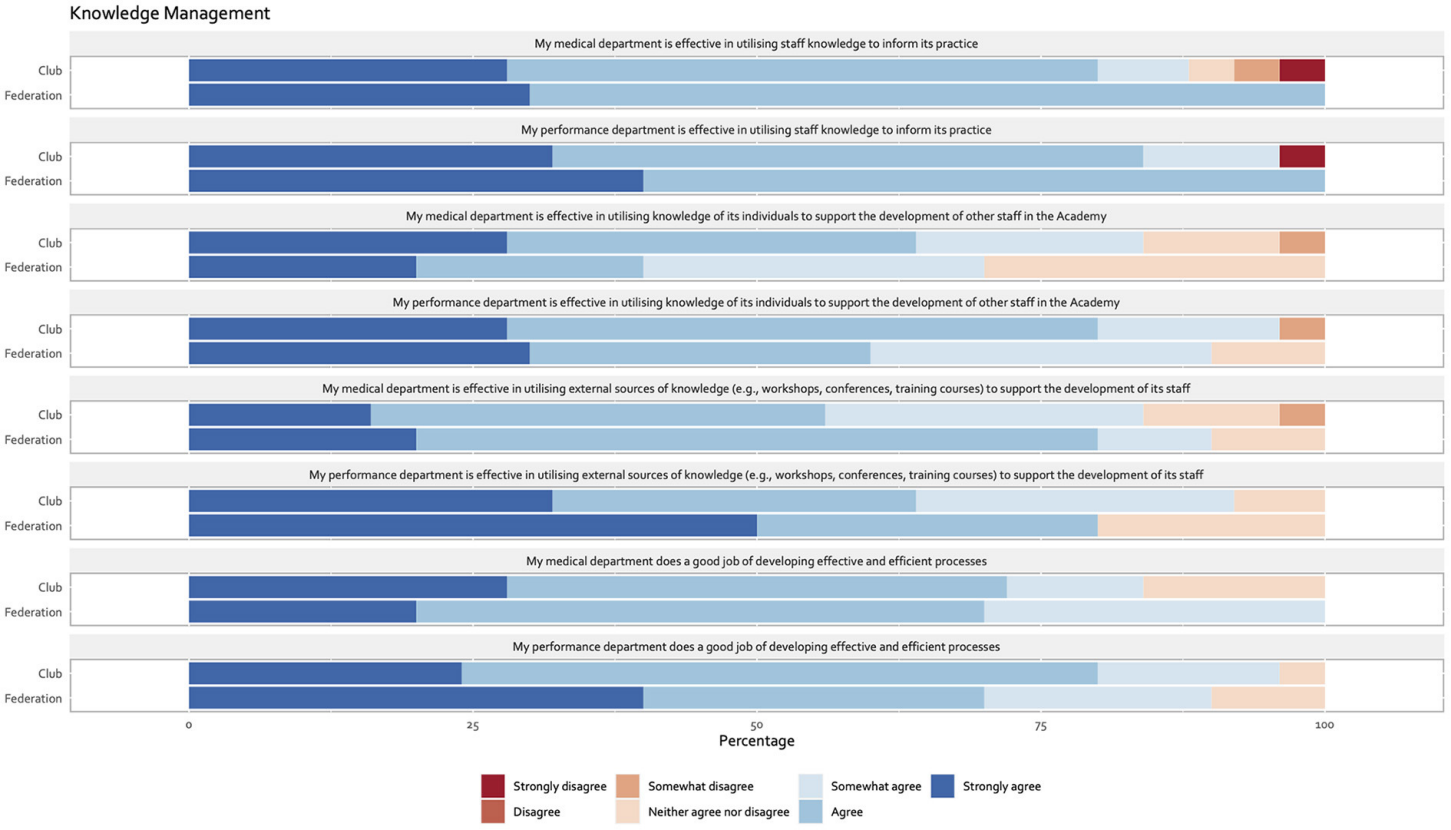
Knowledge management.

Results were summarized and illustrated in Figure 1. The majority of respondents agreed their medical and performance departments were effective in utilizing staff knowledge to inform practice at their clubs and national federations, respectively (Figure 3). The majority of respondents also agreed that their medical and performance departments were effective in utilizing the knowledge of its staff and external sources of knowledge to support the development of other staff in the Academy (Figure 3). However, in national federations, less than half of respondents agreed their medical department was effective in utilizing knowledge of its staff to support the development of other staff in the Academy (Figure 1). The majority of respondents agreed their medical and performance departments were effective in developing efficient processes within their clubs and national federations (Figure 3).

### Return-to-play phases

The proportion of club and national federation staff members involved in the different stages of return-to-play is illustrated in Supplementary Table 3. During the return-to-training phase, 40%−60% of club and national federation respondents perceived the medical area as extremely influential. Approximately a third (30%−36%) of these respondents perceived the performance area as very influential. The role of medical doctors and physiotherapists was perceived as extremely influential and very influential by approximately a half of club (48%−56%) and a third of national federation (40%) respondents. A third of respondents (30%−32%) perceived dedicated return-to-play specialists as very influential in this phase. Perceptions on the influence of massage therapists, team fitness coaches, gym fitness coaches, and sports scientists were unclear (no consensus amongst the distribution of responses).

For the return-to-competition phase, approximately half of club and national federation respondents perceived medical and performance areas as very influential (40%−48%). Team fitness coaches and physiotherapists were perceived as very influential by approximately a third-to-half of club (40%−44%) and national federation (30%−50%) respondents. The role of doctors in this phase was perceived as very influential and extremely influential by club (44%) and national federation (40%) respondents, respectively. Unclear perceptions emerged regarding the influence of massage therapists, gym fitness coaches, and sports scientists during the return-to-competition phase.

For the return-to-performance phase, the majority of club and national federation respondents perceived the performance area as extremely influential (~60%). In contrast, perceptions regarding the role of the medical area were unclear. Team fitness coaches were perceived as extremely influential by approximately half of respondents (40%−48%). The role of doctors in this phase was perceived as somewhat influential and extremely influential by approximately a third of club and national federation respondents (30%−40%), respectively. A minority respondents perceived sports scientists as somewhat influential and extremely influential at their clubs (24%) and national federations (30%), respectively. The influence of other staff members in this phase was unclear. The majority to almost all of the respondents indicated that consensus among staff members is generally sought to inform the return-to-play process (60%−96%), outcomes from layoffs longer than 28 days are formally reviewed (70%−88%) and recorded to guide future practice (60%−88%) by medical and performance staff. Outcomes of this process are shared with performance team, coaches, and academy director in the majority of clubs (72%−88%).

### Injury prevention

Team fitness coaches were perceived as extremely influential in the injury prevention process by approximately a third-to-half of the club (44%) and national federation (40%) respondents. Likewise, approximately a third of club (32%) and national federation (30%) respondents perceived physiotherapists as very and extremely influential in the injury prevention process. A minority to approximately a third perceived gym and dedicated fitness coaches as very influential in the process within club and national federations, respectively. The role of doctors and massage therapists was perceived as not at all or slightly influential in the prevention process irrespective of the organization context.

The majority to almost all of the respondents indicated that decision making during the injury prevention process is informed by consensus among staff members (80%−90%), the injury prevention process is formally reviewed (60%−72%) and recorded to guide future practice (60%−92%) by medical and performance staff. Approximately half of the respondents (40%−48%) indicated that injury data are reviewed on a weekly basis. A majority of club respondents (68% to 88%) indicated outcomes of this process are generally shared with performance team, coaches, and academy director. Approximately half of national federation respondents (40%−50%) indicated injury prevention outcomes are shared with performance team and coaches. Club and national federations respondents indicated knowledge of (clinical) injury history, training/match load management, and strength training as the three most important factors informing injury prevention strategies. Likewise, respondents indicated staff communication, fixture congestion, and time constraints as the three main challenges faced by medical and performance staff for preventing re-injury.

Club respondents indicated the median number of weekly prevention sessions during pre-training, warm-up, in-training, and post-training was 4 (IQR, 2–5), 5 (2–5), 2 (0–3), and 3 (2–4), respectively. The typical length of each these sessions was 15 min. In national federations, the median number of prevention sessions included in a typical week was 3 (2–5) pre-training, 4 (2–6) during warm-up, 3 (2–5) in-session, and 5 (4–6) post-training. A session during each of these phases typically ranged from 10 min (i.e., post-training) to 25 min (i.e., pre-training). Injury prevention programmes are typically delivered both pre- and post-training both at group and individual player level in the majority of clubs (60%−80%). The majority of the respondents also indicated that programmes are included also in group-level warm-up sessions (68%). Approximately half to the majority of national federations include group-level prevention programmes during pre-training and warm-up (50%−60%).

### Data management

Respondents indicated that medical and performance data were generally centralized across the first team and at academy level in approximately half and the majority of clubs and national federations, respectively (range, 50%−72%). Almost all of the clubs and national federations use a data management system (range, 70%−92%), with an off the shelf solution provided by an external company accounting for approximately a third of responses. Medical and performance data are integrated within the same data management system in the majority of clubs (68%), yet only in approximately a third-to-half of national federations (40%). From a medical perspective, measures of external load (i.e., running distances), physical performance assessments, and subjective measures of perceived effort data were the three most important sources of performance data informing medical decision-making processes. From a performance perspective, injury audits, staff communication, and injury history were the three most important sources of medical data informing the performance-related decision-making process.

### Research and development

Activities inherent to research and development were reported for the majority and approximately half clubs and national federations (range, 50%−72%). Irrespective of the context, club and national federation respondents indicated research and development activities to be led, in general, by the head of performance (38%) and team doctor (21%) with no specific research-training at academic level (i.e., PhD degree). The majority of clubs undertake research internally *via* club staff (~100%), and academic collaborations (50%−88%), whereas external consultants and industry partnerships with companies accounted for approximately half of responses. Research and development at national federations generally takes place internally *via* internal staff. Approximately half of national federation respondents indicated external consultants guide research and development processes, with academic collaborations and industrial partnerships accounting for only a minority of responses.

Approximately a third of club respondents indicated ideas suggested by full-time staff members, strategic departmental decisions, and the presence of specific staff members responsible for research and development as very important to identify areas of research and development, with unclear perceptions on the role of external consultants to inform this process. The perceptions of national federation respondents regarding factors relevant to research and development activities at their organizations were unclear, with approximately a third identifying strategic departmental decisions as very important to this process. Presentations and workshops represented approaches adopted to disseminate knowledge developed from research and development activities in the majority and approximately a third-to-half of clubs and national team federations.

## Discussion

The present study explored and discussed general perspectives of practitioners regarding the operational structure and practices of medical and performance units in elite youth football academies from around the world. This survey addresses the dearth of previous research on operational processes central to the functioning of elite academy medical and performance departments. Importantly, our findings provide a general basis for researchers and partitioners to work toward optimizing the functioning of these important areas in elite youth academies.

### Strategy and structure

An organization's structure and operational systems are closely linked to its strategy and the two are deeply intertwined (4, 5). Organizations generally accept that strategic assets include elements such as operational efficiency and knowledge management (4, 5). While previous research explored the general strategy, structure, and operational systems of elite football clubs in European countries (2), our study provided a more contemporary characterization regarding the nature and implementation of processes within medical and performance departments operating in clubs and national federations from around the world. In the majority of clubs and federations taking part in our investigation, the first team and academy medical and performance operations were strategically aligned, with medical staff overseeing academy medical operations reporting to those overseeing the first team (range, 60%−90%). Similarly, almost all clubs and federations indicated that medical and performance strategies were aligned. In practical terms, this suggests a relatively high degree of integration between senior and academy units as well as medical and performance units within the Academy. An integrated approach has been advocated in other sports in an attempt to move away from delivery models based on reductionist multi-specialist systems, where ineffective decision-making arises through a lack of integration and effective communication (11). In elite football, the benefits of an integrated approach appear supported by investigations suggesting teams with high internal communication quality within the medical team and between the medical and performance team experience lower injury rates and high player availability compared to teams with low communication quality (19). Notably, only 40% of club and 50% of federations had the same individual leading both medical and performance areas, however, we were not currently able to ascertain the underlying rationale for adopting such structures and the relative strengths and weaknesses of each approach.

The scrutiny of the structure underlying club and federation academies from our sample followed principles and notions inherent to the organizational structure frameworks described by Steiger et al. (4) and Mintzberg (15). The majority (~60%) of club and national federation respondents indicated their academies' organization “is defined by its standardization. Work is very formalized, there are many routines and procedures, decision-making is centralized, and tasks are grouped by professional departments. Our roles are clearly defined. We have a formal planning process with budgets and audits, (internal or external) and procedures are regularly appraised for efficiency”. These characteristics align with the organizational type described as “strategic business unit” where formalization is important including the development of procedures for many important operating processes (3, 16, 20). Strategy is typically a top-down function with decision-making, strategic planning and work process managed at the top of the organization (4, 15). The remaining club and federation academies respondents largely indicated their academies' organization “has a simple, flat structure consisting of one large unit with one or a few top managers. The Academy is relatively unstructured and informal compared with other types of organizations, and the lack of standardized systems allows the Academy to be flexible and make decisions quickly”. Such organizations are described as functional with greater emphasis placed on the professional skills of the employees alongside increased autonomy (4, 15, 20). The presence of two largely dominant structures supports observations suggesting that, within and between countries, formal structures of modern football organizations are becoming increasingly homogenized (2). This likely represents, to some extent, measures implemented by some key stakeholders (e.g., UEFA) and national football association (2). For example, the more formalized structure of academies in England (21) and an increasing number of European countries (e.g., France and Italy) is likely attributed to the need to align with regulations established by the league governance as part of a centralized funding model to support youth player development. Whilst the benefits and challenges of the two structures presently observed were discussed in the context of other industries (4, 15), understanding how such structures might optimize real-world solutions in the context of the key performance indicators common to the modern football academy could warrant further investigation. Likewise, given the global reach of the current survey, more in-depth research might shed more light on the factors responsible for the dominant structures currently in place at modern football organizations (e.g., governance, strategic, cultural, financial etc).

Within the overall academy structure, part-time contractual staff typically out-numbered full-time staff in both club and federation academies, lending support to previous observations on the overall structure of elite global academies (2). The substantial heterogeneity in the composition and number of medical and performance professionals employed in the present teams aligns with previous findings in a survey of 78 European professional teams (22). Doctors and physiotherapists were present in all club and federation academies, yet the number employed varied especially those on part-time contracts. The variation in the number of massage therapists, sport scientists and pitch-based fitness conditioning staff (team level) was also evident with no part-time or full-time individual employed in these capacities in some academies. Similarly, no part-time or full-time osteopath/chiropractor or psychologist were employed in some club and federation academies. All academies employed at least one part-time dedicated nutritionist within the medical or performance team and one dedicated return to play specialist. A number of factors are likely to explain this diversity in the composition of medical and performance expertise. As previously noted, measures implemented by some national football associations and leagues play an important role in some countries (2). For example, youth development regulations in the English Premier League (21) and Italian Football Federation (23) means it is mandatory for clubs to incorporate specific expertise in accordance with its academy category. In France, the French Football Federation's regional academies are required to include a full-time performance analyst and strength and conditioning coach within their structure. In these countries, external financial support is often provided to the clubs to facilitate the introduction of more specialized staff. In other countries (e.g., Qatar), the national football association and league offer financial support to the clubs along with centralized support encompassing medical and performance expertise. The relative importance of youth development to each club's business model together with the club's own financial resources will also influence the scale and composition of staff employed within the same country and between countries (2). Since a high proportion of clubs and federations indicated the first team and academy medical and performance operations were strategically aligned, it is likely, in some instances, that specialist staff from the first team environment were also providing some support to the Academy where such resource was not available. Aside from any financial benefit, importantly, such integration is only likely to be advantageous in facilitating effective communication across the clubs medical and performance team (11). Different working practices within and between countries due to different practitioners' responsibility, skill base and the specific culture associated with a particular environment are also likely to have influenced the scale and composition of staff available (2).

### Knowledge management

As organizations strive to enhance their competitive advantage in today's knowledge-based economy, knowledge management has become an increasingly important strategic asset for organizational success (4, 24–26). Knowledge transfer is a key component of knowledge management and is concerned with promoting and facilitating knowledge sharing, collaboration, and networking for better decision-making (4, 6, 26, 27). Our study examined four components of knowledge transfer within the context of academy medical and performance setting. The majority of respondents highlighted their medical and performance department was effective in utilizing staff knowledge to inform its practice, utilizing the knowledge of its own staff and external sources of knowledge to support the development of other staff and developing effective and efficient processes for transferring knowledge. Business strategy and organizational structure represent important success factors mediating knowledge management (24). The relatively high degree of integration between senior and academy departments as well as Academy medical and performance departments, together with their largely formalized structures, is therefore likely to have served to some extent to facilitate knowledge transfer within the Academy setting. Interestingly, less than half of respondents in federations agreed their medical department was effective in utilizing knowledge of its staff to support the development of other staff. Since no apparent differences in organizational structures and systems were currently identified between club and federations, further work may be relevant to elucidate the reasons for such differences. Additionally, organizational culture, leadership and technology represent important elements of knowledge strategies for successful knowledge management implementation (6, 28, 29). It is, therefore, likely that several factors beyond the reach of the current study contribute to the reported limitations in knowledge transfer currently observed.

### Return-to-play phases

Return-to-sport (play) decision-making in sport is complex and challenging since it is linked to the athlete's wellbeing and performance (30). A number of frameworks have been developed to facilitate decision-making during return-to-sport (16, 31, 32). The notion that shared decision-making amongst the various disciplines, coach and athlete is likely to improve outcomes and satisfaction with the process by eliminating the contextual “blind spots,” such as an individual's expectation, preference, and value is common to the different framework's researchers proposed to date (11, 30, 31). Accordingly, our study sought to derive insights into decision-making across key stages of the return-to-sport continuum: return to participation (training), return to sport (competition) and return to performance (16). Shared decision-making was common practice with the majority to almost all respondents indicating that decision-making was informed by consensus amongst all medical and performance practitioners involved in the process. Outcomes from layoffs >28 days were formally and collectively reviewed by the medical and performance team, recorded to guide future practice, and shared across with the wider performance team, coaches, and academy director. Alongside shared-decision-making, the present findings also document, for the first time, the relative influence of medical and performance units together with specific roles within each unit are dependent on the stage of the return-to-play continuum. Doctors and physiotherapists were perceived to be very influential to extremely influential during return-to-training and return-to-competition phases, respectively, while the majority of respondents perceived the performance area as extremely influential during return to performance. A dedicated return-to-play specialist was perceived as very influential during return to training while team fitness coaches were seen as very influential during return-to-competition and return-to-performance phases. Sports scientists were also extremely influential during the return-to-performance phase.

### Injury prevention

Injury prevention in the real-world is complex due to the diversity of contextual factors which influence the implementation of injury prevention programmes (33, 34). Research directed toward understanding contextual factors which influence the delivery of injury prevention strategies in professional football academies is limited (35, 36). We sought to derive insights into the delivery, content and implementation challenges of injury prevention strategies in elite academies. Individuals delivering injury prevention programmes play a key role in achieving the desired outcomes (36, 37). Approximately 40% of clubs and federations indicated that team fitness coaches were very influential in the injury prevention process while approximately a third of respondents perceived physiotherapists as very and extremely influential. In some instances, up to a third of respondents also highlighted that gym and dedicated fitness coaches were very influential in the process. Fitness coaches have also previously been identified as the primary deliverers of injury prevention programmes in a small sample of professional academies (36). Consistent with this, the position of the physiotherapist was the most represented position in the injury prevention programme in a survey of professional teams (38). In this context, doctors were perceived as not at all or only slightly influential in the prevention process irrespective of the organization context in contrast to perspectives concerning their importance in the return-to-play process. This may infer input from doctors is mainly strategic in nature with development and delivery of the programme undertaken mainly by other experts in the support team. Indeed, doctors have previously been shown to be mainly involved in the design and assessment aspects of injury prevention programmes with little input into the delivery phase (38).

A range of injury prevention facilitators and barriers have previously been observed in professional football settings (35–39). In line with such observations, club and national federations respondents indicated injury history, training load management, and strength training as three important factors informing injury prevention strategies (39). The majority (60%−90%) of respondents currently reported that decision-making was sought through consensus amongst those staff involved in the injury prevention process. Majority of club respondents and half of national federations also reported outcomes of the process are generally shared with performance team and coaches with clubs also sharing outcomes with the academy director. These insights align closely with observations in professional youth soccer from a delivery and support perspective where programme implementation facilitators included acceptance/support from the head coach and other staff, yet lack of communication and teamwork emerged as important barriers (35, 36). In fact, staff communication was currently rated as one of three main challenges faced by medical and performance staff for preventing re-injury. A lack of planning is also seen as a barrier to successful programme implementation (35, 36). In line with such observations, the majority of club and federation respondents reported that the injury prevention strategy is formally reviewed, recorded to guide future practice by medical and performance staff with nearly half of the respondents indicating that injury data are reviewed on a weekly basis. Club and federation respondents also rated fixture congestion and associated time constraint as two of the three main challenges faced by medical and performance staff for preventing re-injury. Similar club and governing body related barriers have been documented in other youth professional football settings (35, 36).

Programme structure and practicality from a delivery perspective also constitute important facilitators/barriers for programme implementation (35, 36). The median number of injury prevention sessions delivered on a weekly basis by clubs and federations was ~15. In clubs, the majority of sessions were delivered pre-training and during the warm-up period while federations tended to deliver more sessions before and after training. Sessions were typically done as part of a group though the majority of clubs also included programmes at the individual player level. The number of weekly sessions is far higher than previously observed in elite senior professional players with players typically undertaking 2–5 sessions per week during the pre-season and in-season periods (38). Such differences probably reflect the increased time constraints placed upon senior players due to the demands of competition. With less competition demands, especially during the mid-part of the week, there is far more opportunity to allocate time to player development within the youth setting.

### Data management

The ongoing evolution of the football industry in recent years resulted in modern organizations broadening the number of support staff and their internal domain expertise (40). The progressive metamorphosis of football organizations into structured corporates dedicated to optimal player management reflected the impact of embedding sports sciences in pursuing an evidence-based approach *via* the integration of best practice and experience informed by academic research findings (40). In practical terms, this contributed to re-shaping routine processes of contemporary football organizations now recruiting professionals with backgrounds in physiology, strength and conditioning, biomechanics, and performance analysis (40). Nevertheless, the need to develop systematic analysis frameworks to enhance performance within their organizations is a recent phenomenon that received particular consideration. Accordingly, the business-related nature of modern football organizations now demands establishing flexible data analytics solutions to leverage processes for strategic advantage and remain competitive on both a sport and financial levels (41). Starting with a formal understanding of the business strategy (41), integrating all these cyclical elements is necessary to generate more nuanced sources of information that may be deployed into applied business solutions. From an operational standpoint, establishing formal athlete management systems now constitutes the first step for football organizations to turn data into knowledge that can streamline decision-making processes at corporate and practitioner levels (40).

Our findings revealed an appreciation among football organizations for the centralization of information, with this practice more common at a club than national federation level. Conversely, club and national federation respondents indicating an off the shelf solution provided by an external company highlighted a general lack of expertise in deploying information technology solutions and data analysis at their organizations. Such a finding is consistent with the fact that embedding information technology and data analytics expertise into the support staff remains at an embryonic stage, thereby limiting the promotion of decision support services integrating elements of data collection, analysis, and communication at present (40). To advance current service provision processes and cope with the demands of a self-evolving industry, our investigation further highlighted the need for modern football organizations to ensure their support teams are equipped with information technology, biostatistics, and epidemiology expertise (42).

### Research and development

In today's knowledge economy, football organizations must innovate in order to remain competitive in their industry. Innovation *via* applied research is commonly undertaken within professional team sports in attempt to positively impact performance when translated into practice (43, 44). Building on previous observations on collaborative sport science research in professional team sports (44), we sought to better understand the research philosophies of the club academies and national federations. In line with these observations, research activities were reported for the majority of clubs and approximately half the national federations. Overall leadership of research activity was undertaken by senior performance or medical staff within the club or federation despite no specific research-training at academic level (i.e., PhD degree). Whilst research leadership has typically been driven by the academic partner where teams engage in research collaboration with universities, the role of the practitioner is typically more evident in driving the strategic direction of the research vs. the more technical elements of the research (44). Unless sufficiently equipped with research skills, in the current context, it is likely the nature of the role of senior performance and medical staff is more strategic in nature from the perspective of the club or federation. In line with this observation, approximately a third of club respondents indicated that the departmental strategy together with ideas suggested by full-time staff members and the presence of specific staff members (full-time employed) responsible for overseeing research and development as very important to identify areas of research and development. Strategic departmental decisions were also deemed very important to the research process by a third of all federations. This suggests the core internal staff play a key role in driving the research strategy of clubs and federations. Indeed, the role of external consultants in informing the research direction of clubs and federations was unclear.

Alongside leadership and ideation, the majority of clubs and federations indicated that internal staff also play an important role in research delivery. While this research activity is likely to revolve around the less technical aspects of research *per se* (44), their importance may also partly reflect in some cases the growing number of research-active practitioners undertaking research-based continuous professional development (e.g., PhD and professional doctorate programmes) alongside their day-to-day roles in the organization (26, 44). This development effectively enables them to operate as an embedded scientist within the organization therefore increasing its internal research capability (26, 44). As in other industries, collaborative partnerships with external entities such as universities and private entities have also become increasingly popular vehicles through which sports organizations can harness the required knowledge and expertise to drive research activity (26, 43–45). Individual external consultants (e.g., University academics) accounted for half of responses in both clubs and federations. Despite the majority of clubs also engaging in more formal University research collaboration (e.g., PhD student) and industrial partnerships with companies, only a small number of federations engaged in such activities. Any consideration regarding the contribution of the factors explaining such differences cannot be discerned from the present investigation. Nevertheless, it would be plausible to assume that increased access affords greater opportunity to undertake more extensive research programmes since players spend the majority of their time in the club environment.

## Conclusion

The present survey of elite youth professional football academies from around the world provides new insights regarding key operational processes delivered by medical and performance practitioners. The organizational structure of the majority of academies was very formalized and standardized. Strategy was typically a top-down function with decision-making, strategic planning, and operating processes managed at the top of the organization. Academy medical and performance units were strategically aligned as well as closely integrated with their respective first team units. Survey responses indicated the composition and number of medical and performance professionals employed in academies were both heterogenous. Medical and performance units were largely effective in utilizing staff knowledge to inform its practice, utilizing the knowledge of its own staff and external sources of knowledge to support the development of other staff and developing effective and efficient processes for transferring knowledge. During injury prevention and return-to-play processes, shared decision-making was common practice. The relative importance of specific practitioner's during both injury prevention and return-to-play processes was highlighted by the academies. Medical and performance data were integrated within the same data management system in clubs but to a lesser degree in federations. Research and development activity were reported for most academies and typically led by the head of performance or team doctor. The majority of academies conduct research internally *via* club staff, academic collaborations and/or external consultants and industry partnerships. Information regarding practices and perceptions of professional clubs in the aforementioned area will be of interest to those in the football industry. Collectively, the present study provides a contemporary overview which can inform both current practice and future research in football academy medical and performance departments.

## Data availability statement

The raw data supporting the conclusions of this article will be made available by the authors, without undue reservation.

## Ethics statement

The studies involving human participants were reviewed and approved by Aspire Zone Foundation Institutional Review Board, Doha, State of Qatar (protocol number: E202007005). The patients/participants provided their written informed consent to participate in this study.

## Author contributions

WG, CC, AG, JO'B, PR, FT, DB, EL, JM, LL, and VS provided substantial contributions to the conception and design of the work and interpretation of the findings. LL conducted the data analysis. WG and LL wrote the manuscript. All authors read and approved the submitted version.

## Conflict of interest

Authors WG, DB, EL, LL, and VS were employed by the company Aspire Academy. Author AG was employed by the company Juventus FC. Author JO'B was employed by the company Red Bull Athlete Performance Center. Author PR was employed by the company Premier League. Author FT was employed by the company Sporting Clube de Portugal. The remaining authors declare that the research was conducted in the absence of any commercial or financial relationships that could be construed as a potential conflict of interest.

## Publisher's note

All claims expressed in this article are solely those of the authors and do not necessarily represent those of their affiliated organizations, or those of the publisher, the editors and the reviewers. Any product that may be evaluated in this article, or claim that may be made by its manufacturer, is not guaranteed or endorsed by the publisher.
