# Supplementary material for: A survey of organizational structure and operational practices of elite youth football academies and national federations from around the world: A performance and medical perspective
Source: Front Sports Act Living. 2022 Nov 23;4:1031721. doi: 10.3389/fspor.2022.1031721 (PMC9727309; doi:10.3389/fspor.2022.1031721)
Supplement: Supplementary file 1 [file Data_Sheet_1.PDF]

Aspire in the World Fellows Research Survey

Introduction to the Survey

Optimisation of the structure of medical and performance support teams is strategically important for all clubs and sporting organisations. In this context, the term performance refers to roles and processes predominantly involved in the physical development and preparation of the player to compete. The term medical refers to roles and processes predominantly concerned with player health with a particular focus on the treatment and prevention of injury and illness.
With the term sporting "Academy" denoting a system of youth teams involving young football players either in professional clubs or national federations, the purpose of this survey is:

i) to explore the organisational structures and working practices of elite youth academies globally;

ii) to explore perceptions of staff regarding the efficacy of their Academy organisational structure and working practices

The questions in this survey relate only to the medical and performance support provided to male youth players in the Academy. The results could be presented at a conference and published in a peer-reviewed scientific journal. In accordance with the Law No. (13) of 2016 Concerning Personal Data Protection in the State of Qatar, all responses will be anonymised and will be stored securely.

Aspire in the World Fellows Research Survey

Consent Form

Risks / Benefits / Confidentiality Of Data

There are minimal risks that you may feel coerced to participate or anxiety regarding your survey responses. However, the survey responses are confidential and all data will be anonymous. Further, any data used in publication will be de-identified to not show club names and you will not be asked to provide your name. There will not be any costs or benefit from participating, aside from the benefit of contributing information that will further the understanding of practices and perceptions within elite football. A limited number of research team members will have access to the data during data collection.

Participation Or Withdrawal

Your participation in this study is voluntary. You may decline to answer any question and you have the right to withdraw from participation at any time. If you do not want to participate either simply stop participating or close the browser window.

Contacts

If you have any questions about the study or need to update your email address contact the researcher Prof. Valter Di Salvo via email to valter.disalvo@aspire.qa. This study has been Reviewed by the Aspire Zone Foundation (AZF) Institutional Review Board (IRB).

Questions About Your Rights As A Research Participant

If you have questions about your rights or are dissatisfied at any time with any part of this study, you can contact, anonymously if you wish, the AZF IRB/RO (Research Office) by phone at +974 44132647 or email at IRB@aspirezone.qa.

By clicking "Next" you are indicating your consent to participate in the research, if you do not wish to participate please close the browser window.

Aspire in the World Fellows Research Survey

Section A: Personal and demographic information

We recommend one representative from the medical department and one representative from the performance department complete the survey together, but only one staff member's demographic information is required.

* 1. What type of organisation do you work for?

- ◯ Club
- ◯ Federation

* 2. Which country is your organisation headquartered?

(Please specify)

* 3. How many years have you worked in professional football?

* 4. How many years have you worked at your current organisation?

* 5. Current job title at your organisation

6. What is your educational background?

Coaching

Academic

Aspire in the World Fellows Research Survey
Section B: Strategy

* 7. Do the first team and Academy medical operations have a common strategy?

- Yes
- No
- Other (please specify)

* 8. Do the first team and Academy performance operations have a common strategy?

- Yes
- No
- Other (please specify)

* 9. Do those responsible for leading the Academy medical strategy report to those responsible for overseeing the first team?

- Yes
- No

If No, please specify who do you report to?

*** 10. Are the Academy medical and performance strategies led by the same individual?**

◯ Yes

◯ No

If Yes, please specify title and educational background

If No,

who does lead the medical department strategy (please specify title and educational background)?

who does lead the performance department strategy (please specify title and educational background)?

*** 11. Are the Academy medical and performance strategies aligned?**

◯ Yes

◯ No

If No, please specify the reason why not

Aspire in the World Fellows Research Survey

Section C: Structure

* 12. Which of the following describes the underlying structure of the Academy where you are currently employed?

- ◯ My Academy has a simple, flat structure. It consists of one large unit with one or a few top managers. The Academy is relatively unstructured and informal compared with other types of organizations, and the lack of standardized systems allows the Academy to be flexible and make decisions quickly.
- ◯ I work for an Academy that is defined by its standardization. Work is very formalized, there are many routines and procedures, decision-making is centralized, and tasks are grouped by professional departments. Our roles are clearly defined. We have a formal planning process with budgets and audits, (internal or external) and procedures are regularly appraised for efficiency.
- ◯ Within my Academy we rely on highly trained professionals and a high degree of specialization. We primarily employ what might be referred to as “knowledge workers.” Workers are autonomous, so control and decision-making are decentralized. My organization is complex, and there are many rules and procedures.
- ◯ Other (please describe)

* 13. Overall, in your Academy how many performance staff members work in your club academy?

(please specify the number)

14. Of the performance staff members, how many work in the following roles? (please select from the Dropdown menu)

In the case of multiple roles, each staff member can only be counted once; in the case one or more of the following roles belong to the medical staff, please count them in question “15”.

	Full-time	Part-time
Fitness conditioning on the pitch (team level)		
Fitness conditioning dedicated to the gym		
Dedicated Nutritionist		
Dedicated Sports Scientist (e.g., load monitoring and data analysis)		
Psychologist		
Dedicated return to play specialist (player level)		
Other		

For Other, please specify the role

15. Of the following staff members in your Academy, how many work in the following age categories? (please select from the Dropdown menu)

One member can figure in more than one age category

	U15	U16	U17	U18	U19
Fitness conditioning on the pitch (team level)					
Fitness conditioning dedicated to the gym					
Dedicated Nutritionist					
Dedicated Sports Scientist (e.g., load monitoring and data analysis)					
Psychologist					
Dedicated return to play specialist (player level)					
Other					

For Other, please specify the role

* 16. Overall, how many medical staff members work in your club academy?

17. Of the medical staff members, how many work in the following roles? (please select from the Dropdown menu)

	Full-time	Part-time
Doctor		
Physiotherapist		
Osteopath/Chiropractor		
Physiotherapist (return to play)		
Massage therapist		
Dedicated Nutritionist		
Psychologist		
Other		

For Other, please specify the role

18. Of the following staff members in your Academy, how many work in the following age categories? (please select from the Dropdown menu)

One member can figure in more than one generation.

	U15	U16	U17	U18	U19
Doctor					
Physiotherapist					
Osteopath/Chiropractor					
Physiotherapist (return to play)					
Massage therapist					
Dedicated Nutritionist					
Psychologist					
Other					

For Other, please specify the role

*** 19. How many years has the current organisational structures for the performance and medical departments at your club academy been in place?**

*** 20. How often is the organisational structure of the academy performance and medical departments reviewed?**

- On a yearly basis
- Other (please specify)

Aspire in the World Fellows Research Survey
Section D: Knowledge Management

* 21. My medical department is effective in utilising staff knowledge to inform its practice.

- 1 – Strongly disagree
- 2 – Disagree
- 3 – Somewhat disagree
- 4 – Neither agree nor disagree
- 5 – Somewhat agree
- 6 – Agree
- 7 – Strongly agree

* 22. My performance department is effective in utilising staff knowledge to inform its practice.

- 1 – Strongly disagree
- 2 – Disagree
- 3 – Somewhat disagree
- 4 – Neither agree nor disagree
- 5 – Somewhat agree
- 6 – Agree
- 7 – Strongly agree

* 23. My medical department is effective in utilising knowledge of its individuals to support the development of other staff in the Academy

- 1 – Strongly disagree
- 2 – Disagree
- 3 – Somewhat disagree
- 4 – Neither agree nor disagree
- 5 – Somewhat agree
- 6 – Agree
- 7 – Strongly agree

* 24. My performance department is effective in utilising knowledge of its individuals to support the development of other staff in the Academy

- 1 – Strongly disagree
- 2 – Disagree
- 3 – Somewhat disagree
- 4 – Neither agree nor disagree
- 5 – Somewhat agree
- 6 – Agree
- 7 – Strongly agree

* 25. My medical department is effective in utilising external sources of knowledge (e.g., workshops, conferences, training courses) to support the development of its staff

- 1 – Strongly disagree
- 2 – Disagree
- 3 – Somewhat disagree
- 4 – Neither agree nor disagree
- 5 – Somewhat agree
- 6 – Agree
- 7 – Strongly agree

* 26. My performance department is effective in utilising external sources of knowledge (e.g., workshops, conferences, training courses) to support the development of its staff

- 1 – Strongly disagree
- 2 – Disagree
- 3 – Somewhat disagree
- 4 – Neither agree nor disagree
- 5 – Somewhat agree
- 6 – Agree
- 7 – Strongly agree

* 27. My medical department does a good job of developing effective and efficient processes

- 1 – Strongly disagree
- 2 – Disagree
- 3 – Somewhat disagree
- 4 – Neither agree nor disagree
- 5 – Somewhat agree
- 6 – Agree
- 7 – Strongly agree

* 28. My performance department does a good job of developing effective and efficient processes

- 1 – Strongly disagree
- 2 – Disagree
- 3 – Somewhat disagree
- 4 – Neither agree nor disagree
- 5 – Somewhat agree
- 6 – Agree
- 7 – Strongly agree

Aspire in the World Fellows Research Survey
Section D: Performance and Medical Processes
Return to play of the injured player

In this section, the return-to-play phase is broken down into specific sub-phases characterising this process. The return-to-training sub-phase refers to the gradual re-introduction of the injured player from non-contact to resuming full team training. The return-to-competition involves the player's progression of competitive match minutes, whereas in return-to-performance sub-phase the player is deemed meeting the required competition demands.

29. Who is involved in the following processes? (please select from the Dropdown menu)

	Return to training	Return to competition	Return to performance
Doctor			
Massage therapist			
Physiotherapist			
Team fitness coach			
Gym fitness coach			
Dedicated return to play specialist			
Sports Scientist			
Other			

For Other, please specify the role

* 30. What is the influence of the medical area in the return to play process (layoff for more than 28 days)? (please select from the Dropdown menu)

	Medical	Performance
Return to training		
Return to competition		
Return to performance		

31. What is the influence of the following roles in each phase of the return to play process? (please select from the Dropdown menu)

	Return to training	Return to competition	Return to performan[illegible]
Doctor			
Massage therapist			
Physiotherapist			
Team fitness coach			
Gym fitness coach			
Dedicated return to play specialist			
Sports Scientist			
Other			

For other, please specify the role

* 32. Is decision-making during return-to-play informed by consensus amongst all those involved in the process?

- Yes
- No

If No, please specify the reason why not

* 33. When completing a long-term rehab process (layoff for more than 28 days), are the outcomes of this process reviewed?

- ◯ No review
- ◯ A formal review with medical and performance team
- ◯ A formal review with medical and performance team
- ◯ A formal review by an external practitioner

* 34. When completing a long-term rehab process (layoff for more than 28 days), is evidence of the process recorded for learning outcomes to shape and guide future practice?

- ◯ Yes
- ◯ No

35. Are the outcomes shared with other staff members?

(please tick all those that apply)

- ☐ Not shared
- ☐ Performance team
- ☐ Coaches
- ☐ Academy director
- ☐ First team staff
- ☐ Technical/sporting director

Aspire in the World Fellows Research Survey

Section D: Performance and Medical Processes

Injury prevention

36. What is the influence of the following roles in the injury prevention process? (please select from the Dropdown menu)

	Answer
Doctor	
Massage therapist	
Physiotherapist	
Team fitness coach	
Gym fitness coach	
Dedicated fitness coach	

37. Please rank at least 3 important factors informing the injury prevention strategy

1	
2	
3	
4	
5	

38. During a 1-game week, how is the injury prevention process delivered? (please select from the Dropdown menu)

	Session per week	Minutes per session	Group	Individual
Pre-training				
Warm-up				
In-session				
Post-training				

39. Please rank at least 3 important challenges faced by medical and performance staff for preventing re-injury

1

2

3

4

5

* 40. Is decision-making for injury prevention practice informed by consensus amongst all those involved in the process?

- Yes
- No

If No, please specify the reason why not

* 41. Is there a review of the injury data?

- No review
- Weekly
- Monthly
- Annually

* 42. Is the injury prevention process reviewed?

- No review
- A formal review with the medical team
- A formal review with medical & performance team
- A formal review by an external practitioner

* 43. Is evidence of the process recorded for learning outcomes to shape and guide future practice?

- Yes
- No

44. Are the outcomes shared with other staff members?

(please tick all those that apply)

- Not shared
- Performance team
- Coaches
- Academy director
- First team staff
- Technical/sporting director

Aspire in the World Fellows Research Survey

Section D: Performance and Medical Processes

Data management

45. How do you manage performance and medical data in the Academy? (please select from the Dropdown menu)

	Medical	Performance
Centralized at club level (across first team and Academy)		
Centralized at academy level		
Centralized at department level		
Locally stored (personal staff PC)		
No data		

* 46. Do you use a data management system in your academy?

- Yes
- No

If No, please specify

47. If you use a data management system in your Academy, is the system

- ◯ an off the shelf solution provided by an external company (e.g. Excel, Smartabase, Edge 10, SAP)
- ◯ a bespoke system designed internally by your IT department
- ◯ a combination of the previous two solutions

Please provide any relevant detail

Aspire in the World Fellows Research Survey

* 48. Are medical and performance data integrated within the same data management system ?

- ◯ Yes
- ◯ No

If No, please specify (e.g. medical confidentiality)

49. From a medical perspective, what are the 3 most important performance data informing the decision-making process?

1

2

3

50. From a performance perspective, what are the 3 most important medical data informing the decision-making process?

1

2

3

Aspire in the World Fellows Research Survey

Section D: Performance and Medical Processes

Research & Development

* 51. Does your Academy undertake research and development in order to enhance practice?

- Yes
- No

If No, please specify the reason why not ? (e.g., Budget Restrictions, Lack of research expertise, permission from club, No value)

If you answer No this section is complete

52. Which staff member/s is responsible for leading research and development (please specify title and educational background)?

53. Rate the importance of the following factors for the Academy to identify the areas of research and development it pursuing (please select from the Dropdown menu)

	Answer
Ideas suggested by internal staff members (full-time employed)	
Strategic departmental level decision	
Specific staff member (full-time employed) responsible for overseeing R&D	
Consultation with external advisers/part-time employed consultants	
Other	

If Other, please specify

54. How does the Academy undertake research and development? (please select from the Dropdown menu)

	Answer
Internally via club staff	
University research collaboration (e.g. PhD students or professional doctorates)	
Individual external consultants (e.g. University academics)	
Industrial partnerships with companies	
Other	

If Other, please specify

55. How is the knowledge developed from research and development activities disseminated to the academy medical and performance staff? (please select from the Dropdown menu)

	Answer
Presentation	
Workshop/focus groups/discussion forum	
Report/Infographic	
Other	

If Other, please specify

Aspire in the World Fellows Research Survey

Any further comments

56. What are the collective strengths of your academy medical and performance departments?

57. What are the aspects requiring improvement in your academy medical and performance departments?

Aspire in the World Fellows Research Survey

Thank you for completing the survey!
