# Supplementary material for: A survey of organizational structure and operational practices of elite youth football academies and national federations from around the world: A performance and medical perspective
Source: Front Sports Act Living. 2022 Nov 23;4:1031721. doi: 10.3389/fspor.2022.1031721 (PMC9727309; doi:10.3389/fspor.2022.1031721)
Supplement: Supplementary file 2 [file Table_1.DOCX]

| **Supplemental Table 1.** Number of staff members by employment status and age category within professional clubs | | | | | | | | |
| --- | --- | --- | --- | --- | --- | --- | --- | --- |
|  | Employment status | |  | Age category | | | | |
|  | Full-time | Part-time |  | U15 | U16 | U17 | U18 | U19 |
| *Medical staff members* |  |  |  |  |  |  |  |  |
|  |  |  |  |  |  |  |  |  |
| Doctor | 1 (0 to 7) | 2 (1 to 10) |  | 1 (0 to 4) | 1 (1 to 4) | 1 (0 to 4) | 1 (1 to 4) | 1 (1 to 6) |
| Physiotherapist | 4 (0 to 8) | 6 (2 to 17) |  | 1 (1 to 6) | 1 (1 to 6) | 1 (1 to 6) | 1 (1 to 6) | 1 (1 to 3) |
| Osteopath/Chiropractor | 1 (0 to 6) | 1 (0 to 2) |  | 0 (0 to 1) | 0 (0 to 1) | 0 (0 to 1) | 0 (0 to 1) | 0 (0 to 1) |
| Physiotherapist (return to play) | 2 (1 to 10) | 2 (1 to 6) |  | 1 (0 to 2) | 1 (0 to 2) | 1 (0 to 2) | 1 (0 to 2) | 1 (0 to 2) |
| Massage therapist | 0 (0 to 5) | 1 (0 to 7) |  | 0 (0 to 2) | 0 (0 to 2) | 0 (0 to 2) | 0 (0 to 2) | 1 (0 to 2) |
| Dedicated nutritionist | 1 (0 to 2) | 2 (1 to 3) |  | 1 (0 to 2) | 1 (0 to 2) | 1 (0 to 2) | 1 (0 to 2) | 1 (0 to 2) |
| Psychologist | 1 (0 to 4) | 1 (0 to 3) |  | 1 (0 to 2) | 1 (0 to 2) | 1 (0 to 2) | 1 (0 to 2) | 1 (0 to 2) |
|  |  |  |  |  |  |  |  |  |
| *Performance staff members* |  |  |  |  |  |  |  |  |
|  |  |  |  |  |  |  |  |  |
| Fitness conditioning on the pitch (team level) | 5 (1 to 12) | 4 (0 to 12) |  | 1 (0 to 3) | 1 (0 to 3) | 1 (0 to 3) | 1 (0 to 2) | 1 (1 to 2) |
| Fitness conditioning dedicated to the gym | 1 (0 to 7) | 2 (0 to 7) |  | 1 (0 to 2) | 1 (0 to 2) | 1 (0 to 2) | 1 (0 to 2) | 1 (0 to 2) |
| Dedicated nutritionist | 1 (0 to 2) | 2 (1 to 3) |  | 1 (0 to 2) | 1 (0 to 2) | 1 (0 to 2) | 1 (0 to 2) | 1 (0 to 2) |
| Dedicated sport scientist | 1 (0 to 7) | 2 (0 to 7) |  | 1 (0 to 2) | 1 (0 to 2) | 1 (0 to 2) | 1 (0 to 3) | 1 (0 to 3) |
| Psychologist | 1 (0 to 4) | 2 (1 to 4) |  | 1 (1 to 2) | 1 (0 to 2) | 1 (0 to 2) | 1 (0 to 2) | 1 (0 to 2) |
| Dedicated return to play specialist | 1 (0 to 3) | 2 (1 to 5) |  | 1 (1 to 2) | 1 (0 to 2) | 1 (0 to 2) | 1 (0 to 2) | 1 (0 to 2) |
| Summary data are presented as median plus minimum and maximum | | | | | | | | |
