# Supplementary material for: A survey of organizational structure and operational practices of elite youth football academies and national federations from around the world: A performance and medical perspective
Source: Front Sports Act Living. 2022 Nov 23;4:1031721. doi: 10.3389/fspor.2022.1031721 (PMC9727309; doi:10.3389/fspor.2022.1031721)
Supplement: Supplementary file 3 [file Table_2.DOCX]

| **Supplemental Table 2.** Number of staff members by employment status and age category within national federations | | | | | | | | |
| --- | --- | --- | --- | --- | --- | --- | --- | --- |
|  | Employment status | |  |  | | | | |
|  | Full-time | Part-time |  | U15 | U16 | U17 | U18 | U19 |
| Medical staff members |  |  |  |  |  |  |  |  |
|  |  |  |  |  |  |  |  |  |
| Doctor | 2 (1 to 4) | 6 (1 to 7) |  | 1 (1 to 3) | 1 | 1 (1 to 2) | 1 | 1 (1 to 2) |
| Physiotherapist | 4 (2 to 6) | 5 (1 to 7) |  | 2 (1 to 5) | 1 (1 to 2) | 1 (1 to 2) | 1 | 2 (1 to 4) |
| Osteopath/Chiropractor | 0 | 1 |  | 0 | 0 | 0 | 0 | 0 |
| Physiotherapist (return to play) | 2 (1 to 4) | 0 |  | 1 (0 to 2) | 0 | 1 (0 to 2) | 0 | 2 (0 to 2) |
| Massage therapist | 2 (2 to 4) | 6 (4 to 7) |  | 1 (0 to 2) | 1 (0 to 2) | 2 (0 to 2) | 0 | 2 (0 to 2) |
| Dedicated nutritionist | 1 (1 to 3) | 1 |  | 1 (0 to 1) | 0 | 1 (0 to 1) | 0 | 1 (0 to 3) |
| Psychologist | 1 (1 to 3) | 1 (0 to 1) |  | 1 (0 to 1) | 0 | 1 (0 to 1) | 0 | 1 (0 to 3) |
|  |  |  |  |  |  |  |  |  |
| Performance staff members |  |  |  |  |  |  |  |  |
|  |  |  |  |  |  |  |  |  |
| Fitness conditioning on the pitch (team level) | 4 (1 to 10) | 4 (1 to 7) |  | 1 (0 to 2) | 1 (1 to 2) | 1 (1 to 2) | 1 (1 to 2) | 1 (1 to 2) |
| Fitness conditioning dedicated to the gym | 0 (0 to 1) | 0 |  | 0 | 0 | 0 (0 to 1) | 0 | 1 (0 to 1) |
| Dedicated nutritionist | 1 (0 to 5) | 0 |  | 1 | 1 | 1 | 0 | 1 (1 to 3) |
| Dedicated sport scientist | 1 (0 to 2) | 1 (0 to 2) |  | 1 (0 to 1) | 0 | 1 (0 to 1) | 0 | 1 (0 to 1) |
| Psychologist | 1 (0 to 3) | 0 |  | 1 (0 to 1) | 0 | 1 (0 to 1) | 0 | 1 (0 to 3) |
| Dedicated return to play specialist | 2 (0 to 4) | 0 |  | 1 (0 to 1) | 0 | 1 (0 to 1) | 0 | 1 (0 to 4) |
| Summary data are presented as median plus minimum and maximum | | | | | | | | |
