# Supplementary material for: A survey of organizational structure and operational practices of elite youth football academies and national federations from around the world: A performance and medical perspective
Source: Front Sports Act Living. 2022 Nov 23;4:1031721. doi: 10.3389/fspor.2022.1031721 (PMC9727309; doi:10.3389/fspor.2022.1031721)
Supplement: Supplementary file 4 [file Table_3.docx]

| **Supplemental Table 3.** Percentage of responses indicating involvement in each specific phase of the return-to-play process | | | | | | | | | |
| --- | --- | --- | --- | --- | --- | --- | --- | --- | --- |
|  |  | Return to training | |  | Return to competition | |  | Return to performance | |
| Role |  | Club | Federation |  | Club | Federation |  | Club | Federation |
| Doctor |  | 92 | 60 |  | 84 | 60 |  | 52 | 50 |
| Massage therapist |  | 16 | 20 |  | 12 | 20 |  | 4 | 20 |
| Physiotherapist |  | 92 | 60 |  | 84 | 50 |  | 56 | 40 |
| Team fitness coach |  | 68 | 30 |  | 88 | 30 |  | 80 | 50 |
| Gym fitness coach |  | 36 | 10 |  | 44 | 30 |  | 40 | 30 |
| Dedicated return to play specialist |  | 60 | 30 |  | 44 | 40 |  | 48 | 30 |
| Sport scientist |  | 40 | 20 |  | 60 | 30 |  | 60 | 30 |
| The return- to-training sub-phase refers to the gradual re-introduction of the injured player from non-contact to resuming full team training. The return-to-competition involves the player’s progression of competitive match minutes, whereas in return- to-performance sub-phase the player is deemed meeting the required competition demands. | | | | | | | | | |
